# Supplementary material for: Expression of CYP2B6 Enzyme in Human Liver Tissue of HIV and HCV Patients
Source: Medicina (Kaunas). 2023 Jun 27;59(7):1207. doi: 10.3390/medicina59071207 (PMC10385124; doi:10.3390/medicina59071207)
Supplement: Supplementary file 1 [file medicina-59-01207-s001.zip › Supplement S1 (extended methodology).pdf]

Supplement S1. Materials and methods

Inclusion and exclusion criteria

Inclusion criteria were age older than 18, both, male and female, and serologically confirmed diagnosis of HIV and HCV infection. Exclusion criteria were hepatitis of unexplained etiology, autoimmune hepatitis, associated liver diseases, and use of all known drugs or substances that are inhibitors or inducers of liver enzymes (*CYP2B6* and *CYP3A4*) and transporter (*ABCB1*). All patients signed informed consent for participation in the study in accordance with the guidelines included in the Declaration of Helsinki and with approval from the Ethics Committee of the Faculty of Medicine, University of Belgrade.

Liver biopsy

A liver biopsy was performed as part of diagnostic and therapeutic protocols in patients for whom it was indicated. Biopsy was performed using Menghini 14 gauge needle. Each tissue cylinder obtained by biopsy was at least 2 mm wide and at least 20 mm in length. After sampling, the liver tissue was divided into two parts. One part was fixed in formalin and used to determine the stage of liver fibrosis and the grade of liver inflammation. The remaining liver tissue was snap-frozen over liquid nitrogen and stored at a temperature of -80°C until further analyses were performed (qRT-PCR). All samples were collected at the VI and XI department at Clinic for Infectious and Tropical Disease "Dr Kosta Todorovic", UCCS, Belgrade, Serbia, and stored at the Department of Pharmacology, Clinical Pharmacology and Toxicology, Medical Faculty, University of Belgrade, Belgrade, Serbia.

Tissue homogenization and RNA extraction from liver tissue

All procedures were carried out in an RNase-free environment and all solutions were made up using RNase-free water/reagents. CK 14 microtubes were used for homogenization. Each sample was placed on ice. With a metal spatula, each sample was removed from collection tubes and placed into CK14 microtubes. 500 µl of QIAzol Lysis Reagent was added to each tube. CK14 microtubes were then placed in the Minilys® personal homogenizer and homogenized for 20 seconds at 5000 rpm. Homogenate was then centrifuged at 12,000 x g for 10 min at 4°C to remove insoluble material. The supernatant was carefully transferred to a new 2 ml collection tube. Tubes containing the homogenates were placed at room temperature (15–25°C) for 5 min. This step was done to promote the dissociation of nucleoprotein complexes. The next step included adding 100 µl of chloroform per 500 µl QIAzol Lysis Reagent. Microtubes were placed in the vortex for 15 seconds. After resting at room temperature for 2–3 min each microtube was centrifuged at 12,000 x g for 15 min at 4°C. After centrifugation, the sample separates into 3 phases: an upper, clear aqueous phase containing RNA; milky white interphase; and a lower, red, organic phase. The upper aqueous phase was then transferred to clean 2 ml collection tubes. In each microtube 250 µl isopropanol was added and then mixed by vortexing. All the samples were then placed at room temperature for 10 minutes and then placed into a centrifuge at 12,000 x g for 10 min at 4°C. A small white pellet was formed after centrifugation containing the RNA at the bottom of the tube. The liquid was carefully aspirated and the supernatant was discarded. In each sample at least 500 µl of 75% ethanol per 500 µl QIAzol Lysis Reagent was added. Each

sample was then placed in a centrifuge at 12,000 x g for 5 minutes at 4°C. Ethanol supernatant was carefully removed and each sample air dried until the pellet become translucent (roughly 30 min). The RNA pellet was redissolved in the appropriate volume of RNase-free water (20-50 µl, depending on pellet size). For each sample, RNA Clean-Up Protocol was performed according to manufacturer instructions.

cDNA synthesis

All procedures were carried out in an RNase-free environment and all solutions were made up using RNase-free water/reagents and RNaseZAP™ (Sigma-Aldrich) was used for surfaces. For cDNA synthesis, TaqMan® Reverse Transcription Reagents (Thermofisher, Cat No. N8080234) were used. Mastermix included RT Buffer, MgCl2, dNTP mix, RNase Inhibitor, MultiScribe (RT enzyme), and random hexamers. 400ng of RNA was reverse transcribed to produce cDNA, which was then diluted to a concentration of 5ng/µl for use in RT-qPCR. RNA quality and quantity were assessed by spectrophotometry using a NanoDrop ND-1000 spectrophotometer (Thermo Scientific, Waltham, MA, USA).

Real-Time qRT-PCR and data analysis

All reagents were thawed on ice and kept on ice until required. TaqMan Gene Expression Master Mix was twirled gently to mix the components. For each condition to be tested, cDNA + H2O in combination were pipetted into two separate sterile, nuclease-free 0.2 ml PCR tubes (one was used for the test gene and one was used for the housekeeping gene) and mixed well by vortexing gently for 5 seconds. qPCR Mastermix was prepared for each gene to be studied e.g. GAPDH (housekeeping gene) and test gene (CYP3A4, CYP2B6 andABCB1), and added to each sample tube.

Expression via RT-qPCR was analyzed for all genes on 96-well white Hard-Shell™ PCR plates (Bio-Rad, Hemel Hempstead, UK, Cat. No. HSP-9601) using Mastermix (TaqMan® Gene Expression Master Mix; Cat. No. 4369016, Applied Biosystems, Waltham, MA, USA), with each well containing 100ng of cDNA (20µl per well). All samples were run in triplicate. Following sample Mastermix addition, the plate was sealed using optically clear adhesive seal sheets (Thermo-Scientific, Cat. No.: AB-1170), and centrifuged for 1 minute at 1300xg, 4° C to ensure proper mixing of reagents and to remove air bubbles. For the blank wells, Sigma H2O was used together with TaqMan® Gene Expression Assay/2X TaqMan® Gene Expression Master Mix.

The following TaqMan® Gene Expression Assay (Life Technologies) probes were used: CYP2B6: Hs04183483_g1; CYP3A4: Hs00604506_m1; ABCB1: Hs00184500_m1, and glyceraldehyde-3-phosphate dehydrogenase (GAPDH): Hs02758991_g1. Plates quantification was performed using Bio-Rad Opticon Monitor™ Analysis software (version 3.1.32)

Fold changes in gene expression were determined as described by Pfaffl. Fold changes in gene expression were determined as described by Pfaffl (Pfaffl M., 2001). Change above 2 fold and below 0.5 fold was considered increased or decreased gene expression.

Tissue homogenization and RNA extraction from liver tissue, cDNA synthesis, and real-time qRT-PCR were done at the Department of Translational Medicine at the University of Liverpool.

Histopathological and histochemical processing

After being fixed in formalin using standard pathohistological procedures, the tissues were embedded in paraffin blocks. They were then subjected to staining using the conventional hematoxylin-eosin method for subsequent pathohistological analysis. To assess the degree of fibrosis, sections from each block were stained by impregnating reticulin fibers with silver. Subsequently, a contrast staining with hematoxylin was applied. The main histological parameters for assessing liver damage in biopsy material were the stage of fibrosis and the degree of liver inflammation. The level of liver fibrosis was classified based on the METAVIR score, as the absence of fibrosis, the presence of mild, moderate, severe fibrosis, and cirrhosis (stages F0-F4). The degree of inflammation is classified based on the METAVIR score; such as the absence of inflammation, the presence of minimal changes, and light, moderate, and high-intensity inflammation. Data on liver fibrosis and liver inflammation levels were obtained from patients records.

Statistical analysis

Categorical variables are presented as absolute numbers with percentages. Numeric variables were presented as means with standard deviations or medians with 25th–75th percentile according to data distribution. Differences in numerical variables between the two groups were assessed by Student's t-test or a Mann–Whitney test, as appropriate, while ANOVA with LSD as a posthoc test was used for assessing differences between three groups of patients. Categorical data were analyzed using a Chi-square test and Fisher exact test. Correlations were examined by correlation coefficients according to the data scale used in the analyses. The level of significance was set at 0.05. Statistical analysis was performed using the IBM SPSS 21 (Chicago, IL, USA, 2012) package. Data analysis was done at the Department of Medical Statistics & Informatics, Medical Faculty, University of Belgrade, Serbia.
