# Supplementary material for: Expression of CYP2B6 Enzyme in Human Liver Tissue of HIV and HCV Patients
Source: Medicina (Kaunas). 2023 Jun 27;59(7):1207. doi: 10.3390/medicina59071207 (PMC10385124; doi:10.3390/medicina59071207)
Supplement: Supplementary file 1 [file medicina-59-01207-s001.zip › Supplement S2 (clinical data summary).pdf]

Supplement S2. Study population clinical data summary

Clinical data	HCV without therapy	HIV	HCV/HIV	HCV receaving therapy		
				Total	PEG-IFN	DAA
Age*	47.81 (±12.82)	57.22 (±18.46)	49.20 (±10.78)	48.5 (±8.81)	51.29 (±10.44)	45.71 (±6.42)
BMI*	23.18 (±4.09)	23.06 (±2.12)	22.61(±3.94)	25.16 (±2.80)	24.46 (±2.77)	25.98 (±2.84)
Gender female**	43.8% (n=7)	11.1% (n=1)	26.7% (n=4)	42.9% (n=6)	42.9% (n=3)	42.9% (n=3)
Alcohol**	25% (n=4)	11.1% (n=1)	13.3% (n=2)	35.7% (n=5)	42.9% (n=3)	28.6% (n=2)
Smoking**	43.8% (n=7)	33.3% (n=3)	40% (n=6)	42.9% (n=6)	42.9% (n=3)	42.9% (n=3)
AST***	53 (33-62)	78 (56-87)	59 (37-78.5)	86 (61-118.5)	87 (63-107)	85 (41-127.5)
ALT***	83 (40-100.75)	98 (85-115)	89 (69-113)	111 (98-179.5)	115 (98-150)	106 (93-183.5)
CRP***	2.5 (1.3-5.25)	2 (2-3)	3 (1-5.5)	2.7 (2-4)	2 (2-3)	4 (2.1-4.5)

*Mean (±Standard deviation); **Percentage within the group (N); ***Median (25th-75th percentyle);

HCV-hepatitis C virus; HIV-human immunodeficiency virus; HIV/HCV-human immunodeficiency virus patients co-infected with hepatitis C virus;

PEG-IFN-pegylated interferon; DAA-direct-acting antivirals;

BMI – body mass index; AST-aspartate aminotransferase; ALT-alanine transaminase; CRP-C-reactive protein.
