# Supplementary material for: Expression of CYP2B6 Enzyme in Human Liver Tissue of HIV and HCV Patients
Source: Medicina (Kaunas). 2023 Jun 27;59(7):1207. doi: 10.3390/medicina59071207 (PMC10385124; doi:10.3390/medicina59071207)
Supplement: Supplementary file 1 [file medicina-59-01207-s001.zip › Supplement S3 (use of medication).pdf]

Supplement S3.Use of medications in study population

	Infection	ART	HCV therapy		Other medications
			PEG-IFN	DAA	
1.	HIV	EFV+ABC/LAM			ASP 100mg daily<br>BIS 1.25mg daily
2.	HIV	DTG+EMT/TDF			
3.	HIV	DTG+EMT/TDF			
4.	HIV	DTG+EMT/TDF			
5.	HIV	EFV+ABC/LAM			
6.	HIV	DTG+EMT/TDF			
7.	HIV	DTG+EMT/TDF			
8.	HIV	DTG+EMT/TDF			
9.	HIV	DTG+EMT/TDF			
10.	HIV/HCV	DTG+EMT/TDF	no	no	
11.	HIV/HCV	DTG+EMT/TDF	no	no	SIL 420mg daily
12.	HIV/HCV	EFV+ABC/LAM	no	no	
13.	HIV/HCV	EFV+ABC/LAM	no	no	
14.	HIV/HCV	DTG+EMT/TDF	no	no	
15.	HIV/HCV	EFV+ABC/LAM	no	no	
16.	HIV/HCV	DTG+EMT/TDF	no	no	
17.	HIV/HCV	DTG+EMT/TDF	no	no	
18.	HIV/HCV	EFV+ABC/LAM	no	no	
19.	HIV/HCV	EFV+ABC/LAM	no	no	
20.	HIV/HCV	DTG+EMT/TDF	no	no	
21.	HIV/HCV	DTG+EMT/TDF	no	no	
22.	HIV/HCV	DTG+EMT/TDF	no	no	
23.	HIV/HCV	EFV+ABC/LAM	no	no	
24.	HIV/HCV	DTG+EMT/TDF	no	no	LOR 1mg daily
25.	HCV		no	no	SIL 420mg daily
26.	HCV		no	no	
27.	HCV		no	no	
28.	HCV		no	no	
29.	HCV		no	no	
30.	HCV		no	no	
31.	HCV		no	no	
32.	HCV		no	no	
33.	HCV		no	no	
34.	HCV		no	no	
35.	HCV		no	no	
36.	HCV		no	no	
37.	HCV		no	no	
38.	HCV		no	no	
39.	HCV		no	no	
40.	HCV		no	no	
41.	HCV			SOF/VEL	
42.	HCV			SOF/VEL	
43.	HCV		PEG-IFN		
44.	HCV		PEG-IFN		
45.	HCV		PEG-IFN		
46.	HCV			SOF	LOR 1mg daily
47.	HCV			SOF/VEL	
48.	HCV		PEG-IFN		
49.	HCV			SOF	
50.	HCV		PEG-IFN		ASA 100mg daily
51.	HCV		PEG-IFN		
52.	HCV			SOF/VEL	
53.	HCV		PEG-IFN		ASA 100mg daily
54.	HCV			SOF	

HCV-hepatitis C virus; HIV-human immunodeficiency virus; HIV/HCV-human immunodeficiency virus patients co-infected with hepatitis C virus;

ART-antiretroviral therapy; DTG-dolutegravir; EMT-emitricabine; TDF-tenofovir disoproxil fumarate; EFV-efavirenz; ABC-abacavir; LAM-lamivudine;

PEG-IFN-pegylated interferon; DAA-direct-acting antivirals; SOF-sofobuvir; VEL- velpatasvir;

SIL-silymarin; LOR-lorazepam; ASP- acetylsalicylic acid; BIS-bisoprolol;
